# New Insights into the Pros and Cons of the Clinical Use of Vitamin K Antagonists (VKAs) Versus Direct Oral Anticoagulants (DOACs)

**DOI:** 10.3390/nu7115479

**Published:** 2015-11-17

**Authors:** Rick H. van Gorp, Leon J. Schurgers

**Affiliations:** 1 Department of Biochemistry, Cardiovascular Research Institute Maastricht, Maastricht University, PO Box 616, 6200 MD Maastricht, The Netherlands; rick.vangorp@maastrichtuniversity; 2 Nattopharma ASA, 1363 Høvik, Norway

**Keywords:** oral anticoagulants, vitamin K, vascular calcification, coumarin, DOACs

## Abstract

Vitamin K-antagonists (VKA) are the most widely used anticoagulant drugs to treat patients at risk of arterial and venous thrombosis for the past 50 years. Due to unfavorable pharmacokinetics VKA have a small therapeutic window, require frequent monitoring, and are susceptible to drug and nutritional interactions. Additionally, the effect of VKA is not limited to coagulation, but affects all vitamin K-dependent proteins. As a consequence, VKA have detrimental side effects by enhancing medial and intimal calcification. These limitations stimulated the development of alternative anticoagulant drugs, resulting in direct oral anticoagulant (DOAC) drugs, which specifically target coagulation factor Xa and thrombin. DOACs also display non-hemostatic vascular effects via protease-activated receptors (PARs). As atherosclerosis is characterized by a hypercoagulable state indicating the involvement of activated coagulation factors in the genesis of atherosclerosis, anticoagulation could have beneficial effects on atherosclerosis. Additionally, accumulating evidence demonstrates vascular benefit from high vitamin K intake. This review gives an update on oral anticoagulant treatment on the vasculature with a special focus on calcification and vitamin K interaction.

## 1. Arterial and Venous Thrombosis

**Figure 1:**
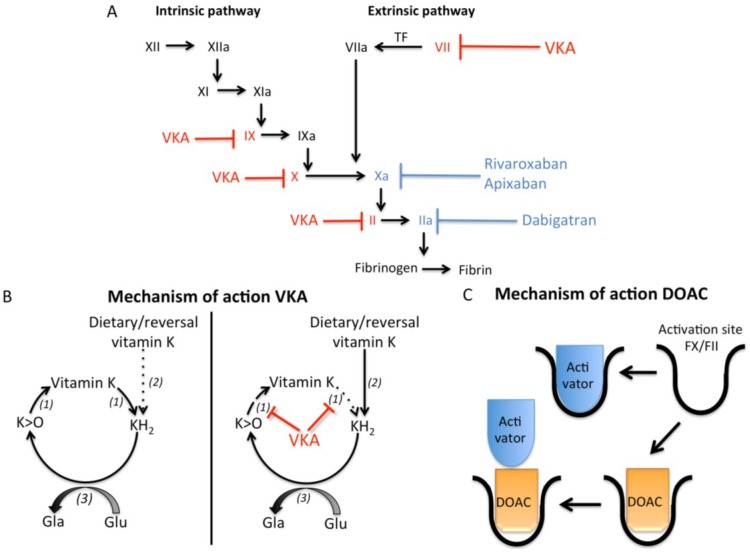
Effects of vitamin K antagonists and direct oral anticoagulants on coagulation. (**A**) The coagulation cascade can be activated by both the intrinsic and extrinsic pathway, which finally results in activation of thrombin and subsequently fibrin formation. Vitamin K antagonists (VKA) induce anticoagulation via inhibiting activation of the coagulation factors depicted in red (factors X, IX, VII, and II). Direct oral anticoagulants (DOACs) induce anticoagulation via blocking the activity of coagulation factors Xa (rivaroxaban and apixaban) and IIa (dabigatran) depicted in blue; (**B**) Vitamin K cycle is required to carboxylate, and thus activate, vitamin K dependent proteins. Vitamin K is converted to vitamin hydroquinone (KH_2_), which is oxidized by γ-glutamylcarboxylase (3) to convert glutamate (Glu) residues in γ-carboxyglutamate (Gla) residues. This reaction results in vitamin K epoxide (K > O), which is recycled to vitamin K through vitamin K epoxide reductase (1). VKA disrupts the vitamin K cycle by inhibiting vitamin K epoxide reductase (VKOR) leading to depletion of vitamin K and uncarboxylated vitamin K dependent proteins. In the liver, the inhibition of warfarin can be circumvented via NAD(P)H quinone reductase (2), which can convert vitamin K into KH_2_ even in the presence of VKA. In extra-hepatic tissues NAD(P)H quinone reductase activity is *ca*. 100 fold less, resulting in inactive vitamin K dependent proteins in the presence of VKA; (**C**) DOACs induce anticoagulation via inhibiting the activity of FXa and FIIa via binding to the activation site.

In 1856 Rudolf Virchow, often regarded as the founder of modern pathology, delineated three major components that were responsible for the formation of emboli in the venous circulation. These three elements, now known as Virchow’s triad, can be briefly summarized as: (1) changes in the composition of blood; (2) alterations in the vessel wall; and (3) disruption of the blood flow. Coagulation is a protective response after vascular injury to prevent bleeding [1] and can be initiated via either the so-called intrinsic or extrinsic pathways, which although simplistic, are still useful schematic models of the coagulation process (Figure 1a). Both pathways are characterized by a series of enzymatic events whereby the activation of members of a hierarchical chain of coagulation enzymes (called coagulation factors) are successively activated by the preceding factor in the chain. Although the initiation steps are different, both pathways converge and lead to activation of pro-thrombin (FII) to produce thrombin (FIIa). An important feature of this coagulation cascade is that it functions as a biochemical amplifier [2] in which the final product, thrombin catalyses the production of fibrin which forms a meshwork clot [3]. The coagulation events leading to the formation of a blood clot (thrombus) that adheres to the wall of a blood vessel and obstructs the flow of blood is termed thrombosis. Thrombosis can take place in both arteries and veins. Atherothrombosis is the term describing the occlusion of a blood vessel by a ruptured atherosclerotic plaque [4,5]. Arterial thrombosis can lead to stroke and myocardial infarction. In contrast to arterial thrombosis, venous thrombosis is associated with dysregulation of coagulation proteins and manifests in deep-vein thrombosis and pulmonary embolism [6]. Obesity and diabetes mellitus are risk factors for both arterial and venous thrombosis whereas other risk factors such as smoking, hypertension and hyperlipidemia increase only the risk for arterial thrombosis [6]. Oral anticoagulant drugs are prescribed to patients to reduce the risk and incidence of both arterial and venous thrombosis, although mainly for the latter.

## 2. The Discovery of Oral Anticoagulant Drugs

The story of the discovery of vitamin K antagonists (VKA) began in the 1920s as a result of an often fatal bleeding disorder in cattle that manifested after the animals had been fed on the hay derived from sweet clover [7]. For this reason the haemorrhagic disease became known as “sweet clover disease”. A crucial observation was the animals that bled had been fed on sweet clover hay that had become mouldy; those animals fed mould-free hay did not present with bleeding [7]. During the subsequent classical studies by Karl Link’s group in Wisconsin it was first shown that bleeding was associated with a low plasma activity of prothrombin as measured by early coagulation assays that were the precursors of the modern day prothrombin time. Link’s group then undertook the task of isolating the haemorrhagic component in spoiled sweet clover that was responsible for the prolonged prothrombin time. This proved a long and arduous process but finally resulted in the isolation and identification of the haemorrhagic agent that we now know as the compound dicoumarol and the first VKA [8]. Dicoumarol is a 4-hydroxycoumarin derivative and originated from microbial action on the compound coumarin which is rich in sweet clover.

After animal trials, dicoumarol was rapidly introduced into the clinic with considerable success. At that time, dicoumarol had also been tested in field trials as a potential rodenticide but it was concluded that the anticoagulant activity of dicoumarol in the rat was not high enough to make it practical for rodent control [8]. Therefore, Link’s group synthesized a large number of different 4-hydroxycoumarin compounds to try and find a derivative with increased anticoagulant activity over dicoumarol. It turned out that a 4-hydroxycoumarin anticoagulant number 42 (out of 150 synthetic variants) was more potent than dicoumarol and had superior pharmacokinetics [8]. This coumarin derivative is today known as warfarin, the word originating from the combination of the Wisconsin Alumni Research Foundation (WARF) and “arin” from coum”arin”. Warfarin revolutionized rodent control but clinicians were reluctant to use a rat poison in humans until an unforeseen event changed their minds. In 1951 an army conscript tried to commit suicide with warfarin. According to Link the slow onset time of warfarin gave him “too much time for thinking” enabling him to reconsider and admit himself to hospital where after receiving vitamin K supplementation he completely recovered. Clinical trials with warfarin in the 1950s confirmed its superiority over dicoumarol, being better absorbed and some 5–10 times more potent, although no head to head comparison was performed. Moreover, it became apparent that an overdose could easily be corrected by vitamin K supplementation [8,9]. Today, warfarin is the most prescribed oral anticoagulant world-wide [10].

### 2.1. Vitamin K and Vitamin K Antagonists

Vitamin K is a fat-soluble vitamin that exists in different forms (Table 1). All forms of vitamin K contain a 2-methyl-1,4-naphthoquinone ring structure, also known as menadione (vitamin K_3_). Phylloquinone (vitamins K_1_) and menaquinones (vitamin K_2_) are classified according to the length and degree of saturation of the aliphatic side chain. Phylloquinone contains a phytyl side chain, and its main dietary sources are green leafy vegetables and certain vegetable oils. Menaquinones have an unsaturated aliphatic side chain comprising a varying number of prenyl units, abbreviated as MK-n (menaquinone with n representing the number of prenyl units). All menaquinones, except MK-4, are produced by bacteria and can be found in fermented foods such as cheese. Additionally, some bacteria in our intestines produce long-chain menaquinones (mainly MK-10 and MK-11) and although they make up the majority of human hepatic reserves their bioavailability for the synthesis of coagulation Gla proteins in the liver is debatable [11]. Both phylloquinone and menaquinones can participate in the γ-glutamylcarboxylation of both hepatic and extra-hepatic vitamin K-dependent proteins, although the longer residence times and better absorption of long chain menaquinones such as MK-7 and MK-9 in the circulation [12,13] makes them more effective for carboxylating both hepatic and extrahepatic vitamin K-dependent proteins [14]. Interestingly, menadione has been shown to act as a endogenous metabolite formed during the *in vivo* conversion of phylloquinone to MK-4 [15]. Furthermore, whereas phylloquinone and menaquinones can reverse VKA induced anticoagulation, menadione per se has no cofactor activity for γ-carboxylation and thus cannot reverse VKA-induced anticoagulation.

**Table 1:** Structural forms of vitamin K.

Drug	Characterization		Dietary Sources
Phylloquinone (vitamin K_1_)	Phytyl side chain		Leafy green vegetables
Menaquinones (vitamin K_2_)	Isoprenoid side chain	MK-4	Meat, eggs
		MK-7	Natto, Cheese
		MK-9	Cheese, curd, sauerkraut
Menadione (vitamin K_3_)	2-methyl-1,4- naphthoquinone		Non-dietary metabolite. Precursor of MK-4

The molecular function of vitamin K is to serve as an essential cofactor to drive the γ-glutamyl carboxylation reaction (Figure 1b). In this vitamin K-dependent reaction, specific protein bound glutamate residues are modified to γ-glutamate residues, hence the name of the γ-carboxylated protein products as vitamin K-dependent proteins (VKDP). To achieve this protein modification vitamin K is first reduced to the active cofactor vitamin hydroquinone (KH_2_) via quinone reductases. Next, the enzyme γ-glutamyl carboxylase (GGCX) oxidizes vitamin KH_2_ with vitamin K epoxide (K > O) as the product. This oxidation reaction is intimately linked and essential to the γ-carboxylation modification of VKDP [16]. The metabolite K > O can be recycled by the microsomal enzyme vitamin K epoxide reductase (VKOR), first to vitamin K and then to KH_2_. This cyclic pathway is called the “vitamin K-epoxide cycle” or simply the “vitamin K cycle” [17,18]. By this salvage mechanism, one molecule of vitamin K is able to carboxylate some 500 glutamate residues [17].

A deficiency of vitamin K can result from an insufficient dietary intake of vitamin K leading to depletion of local vitamin K tissue stores or via interference with the vitamin K cycle by VKA [18,19]. VKA exert their anticoagulant effect by inhibiting the VKOR enzyme resulting in reduced recycling of K > O, thereby limiting KH_2_ production. As the cellular concentrations of KH_2_ decline a stage is reached when the cofactor supply to the GGCX becomes insufficient to fully carboxylate the VKDP that are synthesized in a particular tissue. This in turn leads to the secretion of undercarboxylated species of VKDP in the circulation. The anticoagulant balance exerted by VKA can be said to be ultimately determined by the concentrations of γ-carboxylated coagulation proteins II, VII, IX and X that are secreted into the circulation. Changes in nutritional vitamin K intake interfere with VKA treatment by altering the size of the available pool of KH_2_ cofactor because dietary vitamin K in its quinone state can be converted to KH_2_ by a dehydrogenase enzyme not affected by VKA [18]. Remarkably, despite the long use of VKA the exact mechanism of inhibition of VKOR remains to be elucidated [18].

Owing to their unfavorable pharmacokinetics, VKA have a small therapeutic window, require frequent monitoring, and are susceptible to drug and nutritional interactions. A major disadvantage is that because of their indirect mechanism of action there is a lag phase of 2–3 days before a therapeutic anticoagulant effect is achieved. Therefore, considerable resources have been directed to the discovery of new anticoagulant agents that can directly target specific factors in the coagulation cascade. Several of these so-called direct oral anticoagulants (DOACs) have been approved for clinical use and can be subdivided into agents that either target coagulation factor IIa (FIIa, thrombin) or factor Xa (FXa).

### 2.2. Direct Thrombin Inhibitors

As with the discovery of VKA, the presence in nature of another anticoagulant (albeit in this case an anticoagulant without a lag phase) proved a catalyst to the future discovery of direct inhibitors of coagulation factors. Here it was the isolation of hirudin, a peptide present in the saliva of leeches as a direct inhibitor of thrombin, and long known to prevent coagulation [20,21]. For a detailed review on hirudin, see [22]. Hirudin binds to both the active and substrate recognition sites of thrombin. In addition, it is slowly reversible and excreted predominantly by the kidneys. In the 1990s, recombinant hirudin was shown to prevent postoperative venous thromboembolism [23]. However, a major concern was the increased bleeding tendency with hirudin treatment [21,24]. As with heparin another major disadvantage of hirudin is that it could only be administered by subcutaneous injection. Despite the specific concerns with hirudin, the theory behind designing a direct thrombin inhibitor as a clinical anticoagulant gained support and in the early 2000s a compound called ximelagatran became the first synthetic direct inhibitor of thrombin to be trialed as an oral anticoagulant. However, although initial human trials were promising, later trials led to concerns of hepatotoxicity, which ultimately prevented ximelagatran from being used in the clinical setting [25,26].

The second direct oral anticoagulant to undergo clinical trials was dabigatran etexilate, and this agent received approval for clinical use in 2008 (Table 2). Dabigatran etexilate is a prodrug that requires hydrolysis by carboxylesterases in the body to the active metabolite dabigatran [27]. Dabigatran binds with high specificity to the active site of thrombin thereby inhibiting both bound and free thrombin activity (Figure 1c). It takes two hours before dabigatran etexilate is metabolically active, which eliminates the need for parental anticoagulation. Dabigatran has a half-life of 12–17 h and is usually taken twice daily. Since 80% of dabigatran is excreted by the kidneys, patients with renal problems are not suited for this drug [25,28,29].

**Table 2:** Characteristics of VKA and DOACs.

	VKA [10]			Dabigatran Etexilate [24,27,28]	Rivaroxaban [30,31]	Apixaban [32]
	Warfarin	Acenocoumarol	Phenprocoumon			
**Target**	Vitamin K epoxide reductase	Vitamin K epoxide reductase	Vitamin K epoxide reductase	Thrombin	Factor Xa	Factor Xa
**Pro-drug**	No	No	No	Yes, active metabolite is dabigatran	No	No
**Half-life (hours)**	20–60	8–11	120–144	12–17	5–9	9–14
**Onset time peak effect (hours)**	72–96	36–48	48–72	2	2–3	3
**Duration of action**	2–5 days	<48 h	7–14 days	24–36 h	24 h	24 h
**Metabolism**	Via cytochrome P 450	Via cytochrome P 450	Via cytochrome P 450	Via P-Glucoprotein transporter	Via cytochrome P450 (30%), and P-Glucoprotein transporter	Via cytochrome P450 (15%), and P-Glucoprotein transporter
**Elimination**	Hepatical metabolized	60% Renal	63% Renal	85% Renal	66% Renal	25% Renal
		29% Fecal	33% Fecal	6% Fecal	28% Fecal	
**Bioavailability**	79%–100%	60%	>99%	6.5%	80%	66%

### 2.3. Factor Xa Inhibitors

The idea of using FXa inhibitors as clinical anticoagulants also originates from naturally occurring inhibitors. The first FXa inhibitor to be studied was a compound called antistasin, originally isolated from the Mexican leech *haementeria officinalis* [33]. However, the concept of FXa inhibitors as anticoagulant drugs was not supported until a second FXa inhibitor called the tick anticoagulant peptide (TAP) had been isolated from the soft tick *ornithodors moubata* [34]. *In vitro* and *in vivo* studies demonstrated that FXa inhibitors block the activity of FXa generated via both intrinsic and extrinsic pathways and thus subsequently block the formation of thrombin [35,36].

In 2012 the FXa inhibitor called rivaroxaban was approved for clinical use (Table 2). Rivaroxaban acts via inhibition of the active site of FXa (Figure 1c) [35], and has predictable pharmacokinetic and dynamics [30]. Peak activity of rivaroxaban occurs 2–3 h after intake, with a half-life of 5–9 h [30]. The short half-life suggests that rivaroxaban needs to be taken twice daily, however guidelines for rivaroxaban usage recommend once daily. This recommendation comes from both clinical phase II and III trials, which provided evidence that once daily administration is most beneficial with respect to the balance between safety and efficacy [37]. In addition, the duration of rivaroxaban inhibiting FXa lasts 24 h thereby supporting the once daily policy. Rivaroxaban is mainly excreted by the kidneys (66% with 36% as unchanged drug) with a smaller fraction excreted in the faeces (28% with 7% unchanged) [31].

To date, the most recently approved DOAC is another FXa inhibitor called apixaban (Table 2). Like rivaroxaban, apixaban inhibits both bound and free FXa (Figure 1c). Apixaban activity peaks 3 h after intake and has a half life of 9–14 h [32]. Bioavailability of apixaban is 66%, and apixaban is partly (25%) excreted by the kidneys.

## 3. Clinical Trials with Oral Anticoagulation Drugs

### 3.1. Vitamin K Antagonists (VKA)

The promise and later importance of VKA as oral anticoagulant drugs for clinical use became apparent in a randomized trial performed in the 1960s [38]. In this trial, patients with pulmonary embolism were divided in two groups receiving either the anticoagulant drug or placebo control. Of the group receiving anticoagulation therapy none of the patients died, whereas 5 patients in the placebo group died of pulmonary embolism [38].

Ever since the clinical introduction of VKA, their clinical efficacy and safety have been monitored through measuring the coagulation activity of the blood, mainly using the prothrombin time (PT) test or a close variant of this test [39]. A central ingredient of the PT test is a biological tissue reagent called thromboplastin. It quickly became apparent that innate variations in the source and batch of thromboplastin led to significant variabilities in PT results which were usually reported as a prothrombin time ratio (PTR) representing the patient’s PT divided by normal PT [39]. In principle, when a high or low laboratory PTR is reported, the anticoagulant dosage is adjusted accordingly to reach the target coagulation ratio [39]. In the early 1960s it became apparent that some commercial thromboplastins were insufficiently responsive to the anticoagulant-induced effect leading to an underestimation of the dose of VKA required to achieve the target PTR. The subsequent overdosing with VKA led to an increase in bleeding complications and indicated the importance of using sensitive thromboplastin-based assays to prevent over or under dosing with VKA. Comparison of thromboplastin assays between North America and the UK revealed that increased sensitivity of assays reduced the incidence of hemorrhage [39,40]. These results also addressed the need for increased standardization of PT assays and international guidelines for monitoring anticoagulation therapy. In 1983, the World Health Organization (WHO) adopted a universally standardized system of reporting patient PT data during VKA therapy called the international normalized ratio (INR) which is still used today [41].

### 3.2. DOACs

With the approval of DOACs for clinical use, an alternative to VKA or heparin treatment became available. Recent clinical trials have investigated efficacy and safety profiles of DOACs in comparison to VKA treatment. The outcomes of these comparative trials are briefly described below.

#### 3.2.1. Dabigatran Etexilate

The RE-LY and RE-COVER clinical trials demonstrated non-inferiority of dabigatran etexilate compared to warfarin in atrial fibrillation (AF) patients and patients with acute venous thromboembolism, respectively [25,42]. Additionally, the RE-MODEL clinical trial demonstrated non-inferiority of dabigatran compared to enoxaparin treatment with respect to preventing venous thromboembolism after total knee replacement surgery [43]. Taken together these clinical trials demonstrated the non-inferiority of dabigatran compared to VKA treatment. Thus, dabigatran etexilate seems a suitable alternative for VKA for the treatment of patients with increased thrombosis risk.

#### 3.2.2. Rivaroxaban

The clinical trials ROCKET AF, RECORD, EINSTEIN-DVT and EINSTEIN-Extension demonstrated the non-inferiority of rivaroxaban as compared to VKA and heparin treatment in patients with non-valvular atrial fibrillation, symptomatic venous thromboembolism, deep-vein thrombosis, and recurrent thrombosis in deep-vein thrombosis patients [44,45,46]. Moreover, rivaroxaban demonstrated no difference in risk for major bleedings in patients undergoing elective hip/knee replacement, though rivaroxaban was more effective in preventing symptomatic venous thromboembolism [47].

#### 3.2.3. Apixaban

The ARISTOTLE and ADVANCE clinical trials showed non-inferiority of apixaban as compared to VKA and heparin in AF patients and for thromboprophylaxis in patients after hip replacement, respectively [48,49]. Similar to dabigatran and rivaroxaban, apixaban showed no difference as compared to VKA treatment with respect to risk for major bleedings.

These clinical trials did not demonstrate superiority of DOACs over VKA, questioning whether DOACs should replace VKA as standard treatment. A meta-analysis comparing DOACs with VKA provided additional insight and showed the superiority over DOACs compared to VKA treatment with respect to major bleedings [50]. However, care is required interpreting these results and more research is needed.

## 4. Advantages and Disadvantages of VKA and DOACs

The use of VKA treatment over 60 years is one of the major advantages, since this revealed both short and long-term effects in humans. Disadvantages of VKA include the narrow therapeutic window and thus indirectly safety and efficacy. Therefore, VKA therapy requires regular monitoring by measuring the INR [21,51]. Additionally, the pharmacokinetic and pharmacodynamics are unpredictable through drug interactions, cytochrome P450-dependent mechanisms, and the influence of dietary vitamin K intake [10,52]. All the coumarin VKA listed in Table 2 (*i.e.*, warfarin, acenocoumarol, and phenprocoumon) have a slow onset, taking *ca.* two to seven days to be effective in inducing anticoagulation [10,21]. Therefore, VKA therapy requires initial co-administration with other anticoagulants, such as heparin. Moreover, it has been shown recently that patients taking VKA treatment display hitherto unreported non-hemostasis side effects [53]. One of these side effects is VKA-induced vascular calcification. Vascular calcification is associated with an increased risk for cardiovascular disease [53,54,55]. In addition, VKA treatment is associated with arterial stiffness, which in turn is related to vascular calcification [56,57].

DOACs were developed to circumvent the disadvantages of VKA therapy without negatively influencing safety profiles. Initially, the manufacturers of DOACs promoted the view that a major advantage of DOACs was that they did not require routine coagulation testing (as needed for VKA) or the measurement of circulating drug concentrations. However, data from the dabigatran RE-LY trial suggested that adjusting the dosage of dabigatran based on measurements of plasma dabigatran concentrations may reduce major bleeding events by as much as 30%–40% as compared to well-controlled warfarin [58]. Moreover, monitoring dabigatran plasma concentrations has the potential to improve safety and efficacy profiles as compared to fixed dosage [58,59]. Therapeutic drug monitoring of DOACs is supported by population studies which demonstrated a wide range of plasma dabigatran concentrations in different patient groups and at different time points although drug concentrations remained within the putative therapeutic range [60]. Variations in plasma concentration are ascribed to drug interactions, differences in absorption in the gastrointestinal tract, and clearance by the liver and kidneys [61]. These issues raise the question whether DOAC treatment should be regularly monitored.

A major issue of oral anticoagulation using VKA and DOACs is the need for reversal agents and coagulation assays to monitor the precise degree of anticoagulation. This is of importance in the event of bleeding and to allow surgical procedures or to counteract overdosing. The importance of reversal agents is illustrated by warfarin-related bleeding events, in which 50% of patients die within 90 days, mainly due to intracranial hemorrhage [62]. In the case of VKA, guidelines for the reversal of over-anticoagulation are well established with effective reversal agents (phylloquinone, and prothrombin complex concentrates) and sensitive coagulation assays (e.g., INR) to monitor haemostasis [62]. Vitamin K can counteract the effect of VKA via a NAD(P)H-dependent quinone reductase, the precise identity of which is uncertain. The enzyme that counteracts VKA is predominantly active in the liver and unlike VKOR is not inhibited by VKA thereby enabling the reduction of vitamin K into KH_2_ cofactors needed for γ-glutamyl carboxylation (Figure 1b) [63]. Reversal agents for DOACs were initially lacking, but are currently under development (idarucizumab for dabigatran, and andexanet and PER977 for FXa) [64]. Idarucizumab was developed to inactivate dabigatran, and works by binding to dabigatran with an affinity 350 times higher than thrombin. Recently, the clinical trial RE-VERSE AD investigated the capability and safety of idarucizumab as a reversal agent, and demonstrated a complete reversal of the anticoagulation effect of dabigatran within minutes. Of note, a major limitation in the RE-VERSE AD trial is the lack of a control group [65].

It should be noted that all DOACs are partially excreted by the kidneys (80%, 65%, and 25% for dabigatran, rivaroxaban and apixaban, respectively) [66,67] and are therefore unsuitable for patients with severe renal deficiency. In contrast, VKA are predominantly metabolized through the liver and are thus the best option for anticoagulation with this patient population.

## 5. Vitamin K Dependent Proteins and Atherosclerosis

### 5.1. Coagulation and Atherosclerosis

Coagulation factors are effective activators of the vascular system independent of their effects on coagulation and exhibit pleiotropic effects on the vasculature that contribute to cardiovascular disease. More specifically, coagulation factors can affect the vessel wall through regulation of the proliferation, migration and differentiation of vascular smooth muscle cells (VSMCs) as well as by inducing oxidative stress, inflammation and apoptosis [66,67], all processes that contribute to the development of atherosclerosis. In addition, micro plaque ruptures and subclinical thrombosis are pivotal to the progression and increased vulnerability of plaques, thereby increasing the risk for atherothrombosis [67].

### 5.2. Thrombin and Atherosclerosis

Thrombin is the central player in the coagulation cascade and influences non-hemostasis signaling via the protease-activated receptors (PAR-1, PAR-2, PAR-3, PAR-4) [66]. Thrombin activates these receptors via proteolytic cleavage of the N-terminal domain of PAR-1, PAR-3 and PAR-4 resulting in a tethered ligand activating the receptor [68,69]. In the vasculature, endothelial cells express PAR-1, PAR-2 and PAR-4, whereas VSMCs express PAR-1 and PAR-2 [70]. The effects of thrombin on the different PARs are likely to induce different effects, of which PAR-1 related effects have been most studied [66,71].

**Figure 2:**
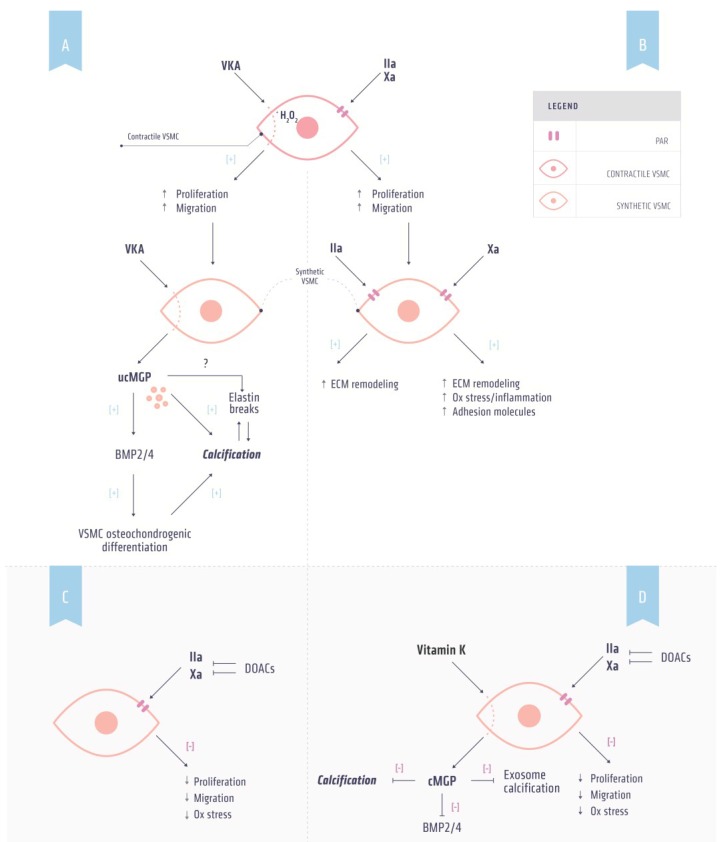
Effects of anticoagulants and vitamin K on vascular calcification. (**A**) Mechanism by which vitamin K antagonist (VKA) induces calcification. VKA induces phenotypic switching of contractile to synthetic vascular smooth muscle cells (VSMCs) by increasing oxidative stress resulting in increased proliferation and migration. Synthetic VSMCs secrete uncarboxylated matrix Gla protein (ucMGP) as a result of vitamin K depletion induced by VKA. ucMGP is unable to inhibit bone morphogenetic protein (BMP) 2 and 4, a marker for osteochondrogenic differentiation. Osteochondrogenic VSMCs are prone to calcification. Additionally, ucMGP is directly associated with increased calcification; (**B**) Thrombin and factor Xa induce non-hemostasis signaling via protease-activated receptors (PARs). Activation of PARs on contractile VSMCs can induce phenotypic switching resulting in increased proliferation and migration. PAR signaling in these synthetic VSMCs increases oxidative stress and adhesion molecules and induces extracellular (ECM) remodeling thereby facilitating calcification; (**C**) DOAC treatment in combination with (**D**) supplemental vitamin K administration has the potential to prevent both hypercoagulability and inhibit vascular calcification.

Human atherosclerotic plaques express elevated levels of PAR-1 [72], suggesting that PAR-1 plays a role in atherosclerosis development. In line with this hypothesis, thrombin induced PAR-1 activation can lead to VSMC migration and proliferation (Figure 2b). Moreover, thrombin elevates collagen production by VSMCs in a PAR-1 dependent manner [73], indicating a possible role for thrombin in plaque stability. Another feature of atherosclerotic plaque development is apoptosis. VSMCs that undergo apoptosis can generate thrombin via a process that accelerates the assembly of the prothrombinase complex [74]. In addition, thrombin generation is associated with vascular calcification [75].

Of note, rodents lack PAR-1 expression on platelets [71]. Therefore, experiments targeting PAR-1 in rodents can be directly ascribed to PAR-1 expressed by the vessel wall. Indeed, vascular injury is altered in PAR-1 deficient mice possibly via extracellular matrix formation and remodeling [72]. In line with these results, *in vitro* stimulation of VSMCs by thrombin stimulates extracellular matrix production [70].

Dabigatran inhibits thrombin mediated PAR1 function by inhibiting N-terminal cleavage and internalization [76]. The inhibition of thrombin activity by dabigatran may thus inhibit the development of cardiovascular disease by reducing pro-inflammatory signaling [67]. Indeed, dabigatran was shown to attenuate atherosclerosis development [77] and promote plaque stability [78].

### 5.3. Factor Xa and Atherosclerosis

Like thrombin, FXa induces non-hemostatic signaling via PARs. However, in contrast to thrombin FXa interacts only with PAR-1 and PAR-2 [70,79]. As with PAR-1, the expression of PAR-2 is upregulated in human vascular lesions [70,80]. FXa has been linked to pathophysiological conditions, including inflammation, tissue fibrosis and vascular remodeling [81]. In line with this link, FXa has been shown to induce inflammatory signaling and increase expression of cell adhesion molecules [67]. Moreover, FXa induces proliferation and migration of VSMCs via activation of PAR-2 thereby altering the composition and accumulation of extracellular matrix [82,83]. The *in vivo* importance of PAR-2 in the inflammation process is suggested by findings that PAR-2 deficient mice display lower inflammation in a model of arthritis [84] and from rat studies in which lipopolysaccharide and oxidative stress increased the expression of PAR-2 [85,86].

Taken together, the available data suggests that FXa has a role in atherosclerosis via its interaction with PAR-1 and PAR-2. Recently, treatment of atherosclerosis prone apoE^−/−^ mice with rivaroxaban resulted in increased plaque stability [87]. Since FXa activates prothrombin it is tempting to speculate that inhibition of FXa also prevents thrombin-mediated effects in atherothrombosis.

## 6. Vitamin K Dependent Proteins and Calcification

Originally, vascular calcification was thought to be a passive process. The discovery of calcification inhibitors that actively prevent vascular calcification showed that it is a highly regulated process involving proteins and cellular components. The VKDP matrix Gla protein (MGP) is a local calcification inhibitor associated with calcifications in human lesions [88,89]. Other VKDP associated with vascular calcification are osteocalcin (OC) and the more recently discovered Gla Rich Protein (GRP) [90]. Moreover, MGP, OC and the downstream regulator bone morphogenetic proteins (BMP) 2 and 4 are associated with microcalcifications in early human atherosclerotic lesions [88]. Microcalcifications in atherosclerosis are associated with increased plaque vulnerability [91,92,93,94].

### 6.1. Osteocalcin

**Figure 3:**
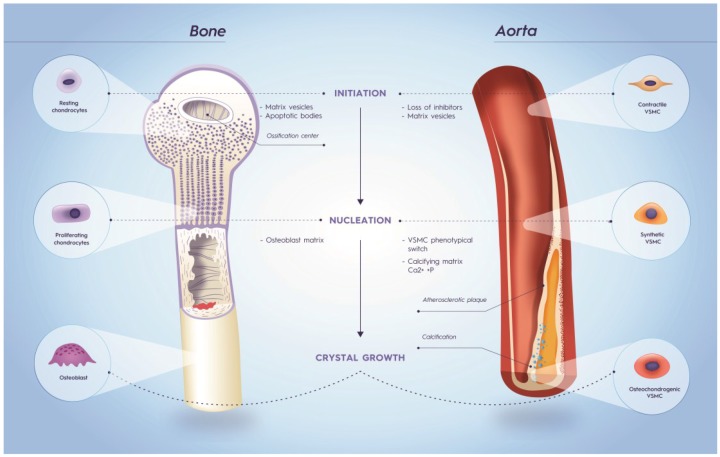
Similarities in bone metabolism and vascular calcification. The calcification process can be divided into three stages: initiation, nucleation and crystal growth. In order to initiate mineralization resting chondrocytes and contractile vascular smooth muscle cells (VSMCs) lose calcification inhibitors. Moreover, vesicles derived from chondrocytes and VSMCs form a nidus for calcification. In both bone metabolism and vascular calcification the matrix plays an important role in the nucleation stage. In bone metabolism, an osteoblast matrix results in proliferation of chondrocytes. Likewise, a calcifying matrix consisting of elastin, collagen and Ca^2+^ and P accompany vascular calcification. Additionally, contractile VSMCs undergo phenotypic switching resulting in synthetic VSMCs, which have increased proliferation and migration in comparison to contractile VSMCs. Finally, osteoblasts and osteochondrogenic VSMCs induce crystal growth in bone metabolism and vascular calcification, respectively.

Like all VKDP proteins, OC requires vitamin K for the γ-glutamylcarboxylation of three glutamate-residues, which in turn confers functional protein activity. In tissues and the circulation OC is present in both the carboxylated (cOC) and uncarboxylated (ucOC) conformations. OC is mostly associated with bone metabolism [95] where it promotes bone growth [96,97,98]. The processes of bone metabolism and soft tissue calcification are closely related, suggesting a possible role of osteocalcin in vascular calcification (Figure 3). As in bone metabolism, OC is thought to both promote [99] and inhibit [100,101,102,103] soft tissue calcification in order to regulate remodeling and mineralization [104]. It has been suggested that the inhibitory effect of OC on calcification is via mechanisms that prevent calcium and phosphate precipitation [17].

The role of OC in vascular calcification suggests the possibility that OC measurements can be used as a biomarker of calcification. OC-positive endothelial progenitor cells are elevated in patients with a history of cardiovascular events and were associated with calcification of coronary arteries [105,106]. During atherosclerosis, VSMCs undergo phenotypic switching resulting in osteoblast-like VSMCs, which are prone to calcification. Calcifying VSMCs express OC [107] and thus increased circulating concentrations of OC may reflect vascular calcification. Indeed, calcification of osteoblast-like VSMCs is associated with OC synthesis [108]. Furthermore, circulating levels of ucOC could provide insights into the relationship between vitamin K status and calcification. Indeed, it has been shown that ucOC concentrations are an independent predictor of carotid artery calcification [109].

### 6.2. Matrix Gla Protein

MGP is found in a wide range of tissues including heart, lungs, skin and the vasculature. In the vessel wall MGP is secreted by VSMCs [110]. Besides posttranslational carboxylation, MGP can also undergo serine phosphorylation. The precise role of the latter modification is not fully understood, but is thought to play a role in the secretion of MGP [111].

The pivotal role of MGP became clear from MGP-deficient mice, which all died within eight weeks after birth due to rupture of severely calcified arteries [112]. Analyses of the arteries revealed fragmented and calcified elastic fibers and the presence of osteochondrogenic-like cells. Surprisingly, the calcification phenotype of MGP^−/−^ mice was not rescued by restoring circulating levels of MGP via hepatic MGP expression [113]. In contrast, restoring MGP expression in VSMCs rescued the vascular phenotype completely [113]. These data demonstrated that only VSMCs synthesis of carboxylated MGP (cMGP) is able to inhibit vascular calcification. MGP deficiency is also present in humans resulting in Keutel syndrome. Patients with Keutel syndrome suffer from abnormal soft tissue calcification [104] and have low levels of circulating cMGP [114].

Administration of warfarin results in similar calcification of arteries as observed in MGP^−/−^ mice suggesting that warfarin-induced calcification is via impairment of MGP function [115]. Additionally, since warfarin chemically knocks down γ-carboxylated MGP it can be used as a model to investigate the role of MGP in vascular calcification [115].

There are several routes by which cMGP may inhibit calcification [110]. MGP is present in matrix vesicles and apoptopic bodies released from VSMCs [116]. In the presence of increased extracellular calcium MGP levels initially increase. However, when calcium levels are chronically elevated, MGP levels decrease [110]. Furthermore, MGP blocks VSMC phenotypic switching. Under physiological conditions, VSMCs display a contractile phenotype supporting vascular tone, and are not prone to calcify. In contrast, VSMCs undergoing synthetic or osteochondrogenic differentiation are susceptible to vascular calcification [17]. MGP^−/−^ mice had decreased VSMC contractile markers and increased osteochondrogenic markers [112,117]. The phenotypic switching of VSMCs is under the regulation of BMP 2 and 4, which in turn are inhibited by cMGP [118,119]. MGP also directly inhibits calcium crystal growth by its ability to block nucleation sites through the binding of the negatively charged Gla domain and phosphorylated serine residues of MGP with growing hydroxyapatite crystals [120,121]. Finally, MGP prevents mineralization of elastin fibers by shedding nucleation sites, this process being facilitated by the low molecular weight and small size of MGP allowing it to prevent mineralization within the elastin fibers [122,123].

### 6.3. Gla Rich Protein

GRP, also termed Upper zone of growth plate and Cartilage Matrix Associated protein (UCMA), is a recently discovered VKDP highly conserved in animals and humans and involved in the inhibition of vascular calcification. Human gene expression and protein accumulation of GRP was shown in the fetal growth plate, vascular system and skin [124]. In human pathological conditions, GRP expression is associated with calcification of skin and arteries [90]. GRP has, like MGP, calcium-binding properties and acts as a calcification inhibitor [125]. The calcification-inhibitory effect of GRP is dose-dependent, requires γ-glutamylcarboxylation, and is thought to act via inhibition of osteochondrogenic switching of VSMCs [125]. GRP knockout mice, however lack phenotypic alterations [126].

## 7. Role for Vitamin K in Cardiovascular Disease

The first evidence that vitamin K is associated with vascular health came from data from the Rotterdam study [127]. In this observational study the risk for cardiovascular disease was some 50% lower in people in the highest tertile for menaquinone dietary intake (MK4 through MK10) [127]. In epidemiological studies, dietary intakes of menaquinones (MK-4 through MK-10) have been reported to be associated with a reduced risk for cardiovascular mortality [127,128]. Additionally, higher menaquinone intakes (MK-4 through MK-10) or supplementation with MK-7 were associated with reduced calcification, which is presumed to be due to the improved γ-carboxylation and greater functional activity of VKDP [129,130]. Finally, the long chain menaquinone isoprenologue MK-7 has been shown to have a greater impact on restoring coagulation compared to phylloquinone in VKA treated healthy volunteers indicating the overall better efficacy of MK-7 for the γ-carboxylation of the coagulation VKDP synthesized in the liver [14,131]. Explanations for the putative beneficial effect of MK-7 on the vascular system for the γ-carboxylation of MGP and GRP is that long chain menaquinones such as MK7 are mainly transported via low-density lipoproteins and have a slower clearance rate from the circulation [13] as well as a higher co-factor activity for the γ glutamyl-carboxylase [132].

In order to investigate the effect of vitamin K on the vascular system in humans a biomarker that reflects vitamin K status is required. Since vitamin K is essential for carboxylation of VKDP, the carboxylation status of VKDP can be used as a biomarker for vitamin K status. Dialysis patients have significantly increased levels of circulating uncarboxylated MGP (ucMGP) and reduced levels of cMGP indicative of a subclinical vascular vitamin K deficiency [133]. Moreover, circulating levels of ucMGP are positively associated with vascular calcification. Taken together, ucMGP seems a promising biomarker for vascular vitamin K status in relation to vascular calcification [89,133,134,135]. Therefore, clinical studies investigating the effect of vitamin K on the vascular system and calcification use the carboxylation status of MGP as a biomarker [130]. Currently, clinical trials are ongoing to assess the effect of high intake of vitamin K on vascular calcification progression [136]. In these clinical studies both phylloquinone and menaquinones are under investigation and can therefore provide novel insights into the differential effect of vitamin K forms on vascular calcification.

## 8. Vitamin K and Direct oral Anticoagulation

Treating patients with hypercoagulability and vascular disease requires personalized medicine. Whereas both VKA and DOACs are equally suitable for treating hypercoagulability, VKA induces vascular calcification thereby affecting the vessel wall in a negative way (Figure 2a). Effects of DOACs on vascular calcification are not known yet, but are unlikely to affect VKDP activity (Figure 2b) [137]. Moreover, high intake of vitamin K has shown to inhibit and even reverse warfarin-induced vascular calcification in experimental animals [56,138] and in adenine treated rats [52]. It is tempting to speculate that co-administration of vitamin K with anticoagulation therapy can target both coagulation and calcification. Since this co-administration is unsuitable with VKA [131], it should be investigated whether combining DOACs and vitamin K can be beneficial for both coagulation and calcification (Figure 2c). Presently, clinical trials assessing these aspects of co-administration are being conducted and the results of these studies should provide novel insights into personalized anticoagulation therapy [136].

## 9. Conclusions

Currently, VKA are still the most widely prescribed drugs used for anticoagulation therapy. However, owing to the unfavorable pharmacokinetics and actions of VKA, direct thrombin and FXa inhibitors have been introduced as alternatives to VKA. Clinical studies have demonstrated that DOACs are non-inferior to VKA but are likely to lack the calcification-inducing side effect of VKA. Additionally, DOACs exert beneficial effects on atherogenesis via PAR signaling. Presently, ongoing clinical trials are addressing whether vitamin K supplementation can halt or regress vascular calcification. The outcome of these trials will pave the way to test whether co-supplementation of vitamin K with DOACs can benefit both coagulation and calcification.
